# Effects and mechanisms of intrauterine chronic hypoxia on ovarian reserve function of zygotic rats

**DOI:** 10.1038/s41598-023-47088-7

**Published:** 2023-11-13

**Authors:** Yanyan Huang, Shanshan Su, Weiwen Luo, Huohu Zhong, Xiali Wang, Guorong Lyu

**Affiliations:** 1 Department of Reproductive Medicine, The Second Affiliated Hospital of Fujian Medical University, Quanzhou, 362000 China; 2 Department of Ultrasound in Medicine, The Second Affiliated Hospital of Fujian Medical University, Quanzhou, 362000 China; 3 Department of Ultrasound, Zhangzhou Hospital, Zhangzhou, 363000 Fujian Province China; 4 Collaborative Innovation Center for Maternal and Infant Health Service Application Technology of Education Ministry, Quanzhou Medical College, Quanzhou, 362000 China; 5 Department of Clinical Medicine, Quanzhou Medical College, Quanzhou, 362000 China

**Keywords:** Intrauterine growth, Organogenesis

## Abstract

Chronic intrauterine hypoxia (ICH) may lead to permanent alterations in the offspring's body structure, function, and metabolism through the "developmental programming" pathway, resulting in lasting changes in physiology and metabolism, as well as the onset of adult-onset diseases. The aim was to investigate intrauterine growth restriction caused by ICH and its effect on ovarian reserve function in female offspring at different developmental stages after birth. Healthy female Sprague–Dawley rats (n = 20) were pregnant by normal mating, and the rats in the ICH group were treated with chronic intrauterine hypoxia twice a day for 04 h00 each time from day 4 to 21 of gestation. After the first hypoxic treatment, four pregnant rats were randomly selected from the ICH and natural control groups for arterial blood gas analysis. In the ICH group, birth weight and body weight on the 5th day after birth were less than in the control group, the total number of follicles and the number of primordial follicles in the offspring of the ICH group were significantly reduced on postnatal days 5, 20, and 40 (*p* < 0.05). ICH decreases ovarian reserve function in female offspring rats and programmatically regulates the differential expression of ovarian miRNAs in female offspring rats.

## Introduction

Chronic intrauterine hypoxia (ICH) is one of the most commonly observed pregnancy complications. Studies have shown that intrauterine hypoxia, among other conditions, may persistently alter the structure, function, and metabolism of the body through the ‘developmental programming’ pathway, leading to permanent changes in both physiology and metabolism and the onset of diseases in adulthood^1–4^. The developing female reproductive system is particularly vulnerable to adverse intrauterine environments because this period is a specific developmental window for building an ovarian reserve. Ovarian reserves are key determinants of fertility^5^ and are largely dependent on the number and quality of the limited number of primordial follicles in the ovary. Disruption of the foetal environment during the critical stage of follicular development can decrease ovarian reserve function in adulthood^6–9^. The weakening of the ability of the ovary to produce eggs, decrease in follicle quality, and corresponding sex hormone changes are known as diminished ovarian reserve (DOR), which can lead to menstrual disorders, infertility, and the further development of premature ovarian failure, seriously affecting reproductive health and quality of life^10,11^. Intrauterine growth restriction caused by maternal malnutrition leads to DNA damage in foetal oocytes of rodents and sheep^12^ and affects follicular development^13^. Engelbregt et al.^14^ constructed an intrauterine growth restriction model by ligating the uterine artery on day 17 of gestation in maternal female rats and demonstrated that intrauterine growth-restricted offspring had delayed puberty and reduced follicle counts in adulthood. The ICH model is a novel experimental model designed to investigate the impact of chronic prenatal hypoxia on the ovarian reserve function of offspring rat. The choice of this model is because chronic intrauterine hypoxia is relatively common during pregnancy, yet its specific effects on the ovarian reserve function of offspring rat have not been fully elucidated.

The exact mechanism of DOR remains unclear, but the hyperactivation and early depletion of primordial follicles as mechanisms leading to DOR have been supported^15^. Thanatsis et al.^16^ used a quantitative reverse transcription-polymerase chain reaction and real-time polymerase chain reaction to detect mRNA levels in the ovarian cortical tissues of five DOR human cases and compared them with those of the control group. They found that the expression of Foxo3a, FOXL2, and p27 mRNAs in the ovarian tissues of patients with DOR were significantly lower than those in the control group, suggesting that Foxo3a, FOXL2, and p27 play a role in the pathogenesis of human DOR. Pampanini et al.^17^ found that intrauterine growth restriction resulted in 24 pairs of genes regulating proliferation, apoptosis, and metabolic cellular functions that were permanently altered in offspring at all developmental stages; however, no studies have investigated whether chronic hypoxia during pregnancy regulates the ovarian reserve function of offspring via the modulation of miRNAs and the mechanism of regulation. Digital gene expression profiling technology refers to the use of high-pass sequencing to directly detect and analyse miRNAs produced by a species or a specific cell in a specific functional state and to study the differential expression of genes^18^. Digital gene expression profiling technology is widely used in basic medicine-related research, diagnosis, treatment, and other fields. The application of digital gene expression profiling technology can assist researchers in identifying miRNAs associated with hypoxia and further investigate how these miRNAs may be involved in regulating the ovarian reserve function in offspring rat.

This study aims to establish an ICH model to investigate the impact of chronic prenatal hypoxia on the ovarian reserve function of offspring rat. Additionally, it employs digital gene expression profiling technology to identify differential miRNA expression, providing robust evidence for elucidating the regulatory mechanisms of hypoxia on the ovarian reserve function in offspring. While there have been some studies on the effects of hypoxia on offspring health, our understanding of the specific molecular mechanisms involved remains limited. Therefore, the innovativeness of this study lies in its utilization of modern molecular biology techniques to delve deeper into this issue, offering further scientific insights into the regulatory mechanisms of chronic intrauterine hypoxia on the ovarian reserve function in offspring.

## Results

### Comparison of biochemical indices after maternal hypoxia in ICH and control groups

**Figure 1:**
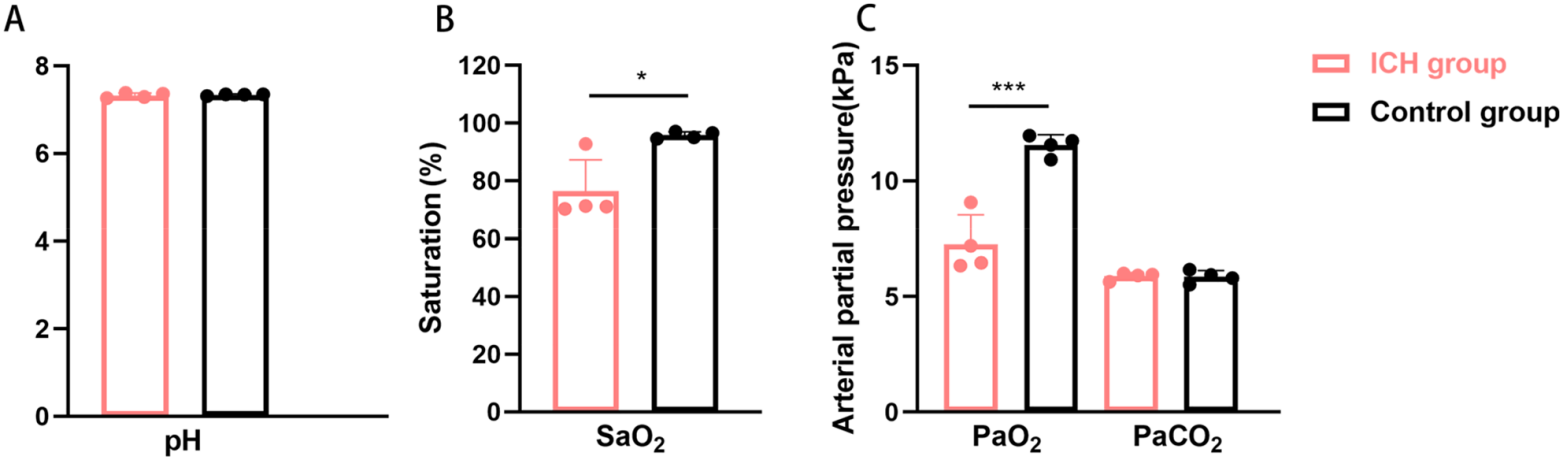
Comparison of blood gas analysis in the ICH and control groups (n = 4 and n = 4, respectively). (**A**) Hydrogen ion concentration index (pH); (**B**) blood oxygen saturation (SaO_2_); (**C**) partial pressure of carbon dioxide (PaCO_2_) and partial pressure of oxygen (PaO_2_). Data are expressed as mean and standard deviation. *p* values were calculated using unpaired t test. *:*p* < 0.05; ***:*p* < 0.001. ICH: intrauterine chronic hypoxia.

The results of arterial blood gas analysis of both groups of pregnant rats after 04h00 of hypoxia demonstrated that the differences in arterial blood pH (7.32 ± 0.06) and partial pressure of carbon dioxide (PaCO_2_) (5.88 ± 0.16 KPa) in the ICH group during gestation (*p* = 0.915 and *p* = 0.915) and in the control group during gestation (*p* = 1.634 and 1.915, respectively) were not statistically significant (*p* = 0.634 and 0.915) (Fig. 1A and C).

Arterial oxygen saturation (SaO_2_) in pregnant rats in the ICH group (76.33 ± 10.92 KPa) was significantly lower than that in the control group (95.80 ± 1.19 KPa) (*p* = 0.012) (Fig. 1B). The partial pressure of arterial oxygen (PaO_2_) in pregnant rats in the ICH group (7.27 ± 1.25 KPa) was significantly lower than that in the control group (11.54 ± 0.45 KPa), (*p* < 0.001) (Fig. 1C).

### Grouping of offspring rat and analysis of birth weight

In this study, the rats under the intrauterine growth retardation model produced a total of 142 offspring rats, including 75 females and 67 males, while the control group produced 87 offspring rats, consisting of 41 females and 46 males. The average BW of female offspring rat in the hypoxia group was 5.76 ± 0.31g, whereas it was 6.77 ± 0.29g in the control group. Therefore, female offspring rat in the hypoxia group with BW below the average BW of the control group by more than two standard deviations, specifically lower than 6.19g, were included in the ICH group, totaling 61 individuals (81.3%). Using a random number table, 30 female offspring rat were selected from both the ICH group and the NC group, dividing them into six subgroups: ICH-A group, ICH-B group, ICH-C group, Control-A group, Control-B group, and Control-C group. Offspring rat that were not included in the study, both female and male, were euthanized by cervical dislocation

### Comparison of weight and ovary size of female rats in the offspring of ICH and control groups

The mean BW was significantly lower in the ICH group at postnatal day 5, and the offspring BW of the ICH group (9.00 ± 1.48 g) was 26% lower than that of the offspring of the control group (12.14 ± 1.41 g), with a statistically significant difference (t = 4.86, *p* < 0.05). However, the offspring showed ‘catch-up growth’ during the lactation period, and there was no significant difference in BW between the two groups on the 20th day of life (50.60 ± 10.05 g in the control group and 49.66 ± 12.50 g in the ICH group, *p* < 0.05). There was no significant difference in ovarian weight (OW) between the ICH and control groups at 5, 20, and 40 d after birth. On postnatal day 5, the OW:BW ratio of female offspring was higher in the control group than in the ICH group, and the difference was statistically significant (t = −2.95, *p* < 0.05) (Table 1).

**Table 1:** BW, OW, and OW:BW of female rats in the offspring of control and ICH groups (n = 10, $\overline{{\text{x}}}$ ± s).

	5 days postpartum		20 days postpartum		40 days postpartum	
	ICH group	Control group	ICH group	Control group	ICH group	Control group
BW (g)	9.00 ± 1.48*	12.14 ± 1.41	49.66 ± 12.50	50.60 ± 10.05	144.67 ± 21.59	152.32 ± 15.13
OW × 10^−3^ (g)	1.80 ± 0.34	2.02 ± 0.53	15.09 ± 3.81	15.09 ± 14.81	38.71 ± 13.64	44.88 ± 9.64
OW:BW × 10^−3^ (g/g)	0.20 ± 0.02*	0.17 ± 0.03	0.30 ± 0.05	0.30 ± 0.05	0.26 ± 0.06	0.29 ± 0.05

ICH: chronic intrauterine hypoxia; BW: body weight; OW: ovarian weight; compared with control group, *p < 0.05.

### Morphological changes in ovarian tissue, follicle counts, and extrapolated ovarian volume were observed under a light microscope in all groups of zygotic female rat

**Figure 2:**
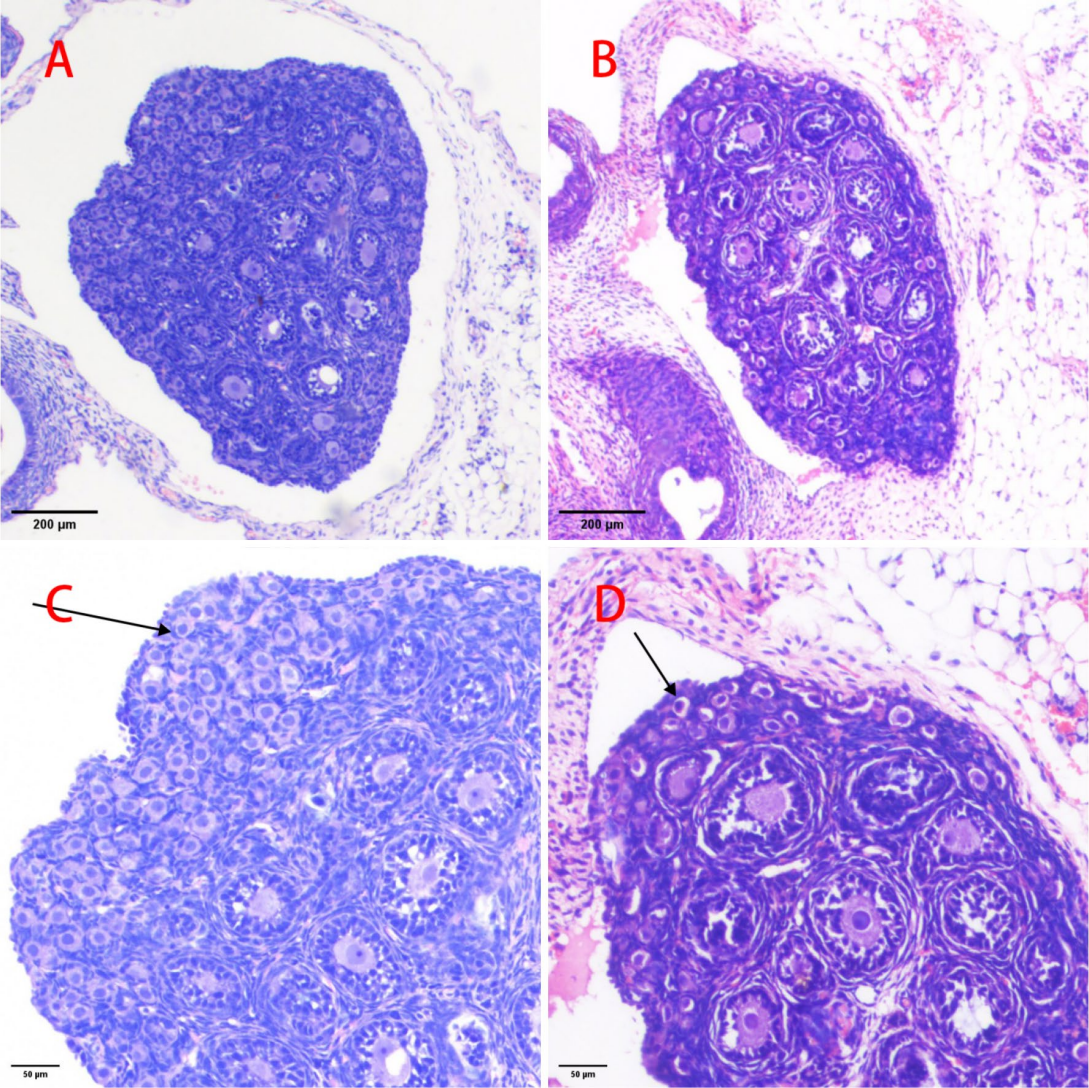
Representative images from a light microscope of ovarian tissue of offspring female rats. (**A**) control group, ovarian histomorphology (HE, ×40); (**B**) ICH group, ovarian histomorphology (HE, ×40) (**C**) control group, representative area with primordial follicles, showing more primordial follicles and relatively dense follicles (arrowheads point to primordial follicles; HE, ×100); (**D**) ICH group, representative area with primordial follicles, showing fewer and sparser primordial follicles than in the control group (arrowheads point to primordial follicles; HE, ×100).

The ovaries of the postnatal day 5 offspring of female rats contained many primordial and primary follicles, as well as some early secondary follicles. Most primordial follicles are located in the cortical portion of the ovary, and most primary and secondary follicles (referred to as growth follicles) are located in the middle of the ovary. The number of follicles at all levels was significantly reduced in the ICH group (*p* < 0.05) compared with those in the control group, but there was no significant difference in morphology (Fig. 2A and B). The number of primordial follicles (n = 3477 ± 358) was 27.1% (t = 6.882, *p* < 0.001) lower than that of the control group (n = 4771 ± 474), and the total number of follicles (n = 3693 ± 362) was 28.9% (t = 7.686, *p* < 0.001) lower than that of the total follicles in the offspring of the control group (n = 5195 ± 501), respectively (Table 2).

**Table 2:** Comparison of follicle counts and ovarian volume in offspring female rats: 5, 20, and 40 days (n = 10, $\overline{{\text{x}}}$ ± s).

Variate	5 days postpartum		20 days postpartum		40 days postpartum	
	ICH group	Control group	ICH group	Control group	ICH group	Control group
Primordial follicle (n)	3477 ± 358***	4771 ± 474	1714 ± 323***	2857 ± 405	980 ± 216*	1678 ± 214
Primary follicle (n)	154 ± 44***	316 ± 70	661 ± 164***	1237 ± 187	770 ± 279	822 ± 237
Secondary follicle (n)	62 ± 11***	108 ± 9	268 ± 35***	491 ± 118	493 ± 189	507 ± 190
Sinusoidal follicle (n)	0	0	268 ± 35***	490 ± 112	421 ± 150	468 ± 165
Total follicles (n)	3693 ± 362***	5195 ± 501	2911 ± 414***	5076 ± 536	2664 ± 510*	3475 ± 460
Growing follicle (n)	216 ± 45***	424 ± 74	929 ± 170***	1728 ± 183	1263 ± 342	1329 ± 348
primordial follicle (%)	94.11 ± 1.33	91.82 ± 1.33	58.70 ± 4.72	56.17 ± 3.48	37.31 ± 8.63	48.78 ± 7.08
Growing follicle (%)	5.891 ± 1.33	8.185 ± 1.33	32.04 ± 4.74	34.15 ± 2.91	47.14 ± 7.68	37.85 ± 6.29
Ovarian volume (mm^3^)	0.034 ± 0.003*	0.039 ± 0.004	14.7 ± 2.88***	20.46 ± 2.44	52.22 ± 2.92*	59.59 ± 6.70

ICH: chronic intrauterine hypoxia; compared with the control group, *p < 0.05; ***p < 0.001.

Antral follicles began to appear in the ovaries of female offspring on postnatal day 20, and the number of follicles at all levels was significantly reduced in the ICH group compared with that in the control group (*p* < 0.05), with no significant difference in morphology. The number of primordial follicles in the offspring of female rats in the ICH group (n = 1714 ± 323) was 40.0% lower than that of female rats in the control group (n = 2857 ± 405) (t = 6.978, *p* < 0.001), and the number of total follicles (n = 2911 ± 414) was 42.7% lower than that of total follicles in the offspring of female rats in the control group (n = 5076 ± 536) (t = 10.107, *p* < 0.001; Table 2).

On postnatal day 40, many well-developed follicles, slightly larger and more structurally defined, were observed in all groups, and there were no significant differences in follicular morphology at any level (Fig. 2C and D). However, the number of primordial follicles in the offspring females of the ICH group (n = 980 ± 216) was 41.6% lower than that in the offspring females of the control group (n = 1678 ± 214) (t = 7.257, *p* < 0.001), and the total number of follicles in the ICH group (n = 2664 ± 510) was 23.3% lower than that of the offspring females of the control group (n = 3475 ± 460) (t = 3.735, *p* < 0.05). No significant differences in the number of follicles were observed at the remaining levels (Table 2).

The extrapolated ovarian volume was significantly smaller in the ICH group than in the control group at 5, 20, and 40 d postnatally (*p* < 0.05; Table 2).

### Digital gene expression profiling

Digital gene expression profiling was used to detect differences in miRNA expression profiles in the ovarian tissues of female rats in the chronic in utero ICH and control groups on postnatal days 5, 20, and 40.

In this study, the expression of 96 genes was analysed; 11 genes were differentially regulated in the ovarian tissues of the ICH and control groups (Table 3). Among those 11 genes, five genes had significantly higher expression and six genes had significantly lower expression in the ICH group than those in the control group; these genes are involved in cell proliferation, regulation, apoptosis, and ovarian vascularisation. Three genes were involved in cell proliferation, survival, and cycle regulation in female hypoxic offspring in the ICH group compared with that in the control group at postnatal day 5. These characteristics are the markers of proliferation Ki-67 (MKi-67), proliferating cell nuclear antigen, and thymidine kinase-1 (Tk-1), and topoisomerase II alpha (Top2) expression was downregulated on postnatal days 20 and 40. Thymidine kinase-1 (Tk-1) expression was also downregulated on postnatal day 40.

**Table 3:** Digital gene expression profiling.

Gene function	Genetic locus	5 days postpartum		20 days postpartum		40 days postpartum	
		ICH group	Control group	ICH group	Control group	ICH group	Control group
Hyperplasia	Mki-67	1.5 ± 0.3↑	1.0 ± 0.3				
	Tk-1	1.6 ± 0.4↑	1.0 ± 0.4			0.4 ± 0.1↓	1.0 ± 0.2
	Pcna	1.3 ± 0.6↑	1.0 ± 0.1				
	Top2a			0.4 ± 0.1↓	1.0 ± 0.1	0.7 ± 0.1↓	1.0 ± 0.3
Vascularisation	PDGFR-α	0.6 ± 0.1↓	1.0 ± 0.1				
	TGF-β2			1.4 ± 0.1↑	1.0 ± 0.1		
	bFGF	0.8 ± 0.1↓	1.0 ± 0.2				
Apoptosis	Casp-3	1.1 ± 0.2↑	1.0 ± 0.5				
	Casp-9					0.3 ± 0.1↓	1.0 ± 0.7
Regulation	IGF1-R					0.8 ± 0.1↓	1.0 ± 0.2
	IGFBP-3					1.3 ± 0.1↓	0.8 ± 0.6

ICH: chronic intrauterine hypoxia; compared with control group, ↑: gene expression was up-regulated; ↓: gene expression was down-regulated.

Three genes related to ovarian angiogenesis were dysregulated in female rats of the ICH group offspring compared with those of the control group offspring, and on postnatal day 5, platelet-derived growth factor receptor alpha (PDGFR-α) and basic fibroblast growth factor (bFGF) expression was downregulated. Fibroblast growth factor (bFGF) expression was also downregulated. By contrast, the expression of transforming growth factor beta 2 (TGF-β_2_) was upregulated on postnatal day 20.

The expression of the gene encoding cysteinyl aspartate-specific proteinase-3 (Casp-3), a notable protein in the apoptotic cascade, was upregulated in female rats of the ICH group compared with those of the control group on postnatal day 5, and the expression of the gene encoding the critical protein in the apoptotic cascade was significantly downregulated in female rats of the ICH group on postnatal day 40. Expression of the gene encoding cysteinyl aspartate-specific proteinase-9 (Casp-9), an important protein in the apoptotic cascade, was significantly downregulated on postnatal day 40.

In addition, the expression of insulin-like growth factor binding protein-3 (IGFBP-3) was upregulated in the offspring of female rats in the ICH group compared with those in the control group on postnatal day 40, and the expression of insulin-like growth factor-1 receptor (insulin-like growth factor 1 receptor (IGF1-R) decreased, which suggests the downregulation of IGF signalling in the ovaries of female rats in the ICH group offspring.

### Measurement of serum AMH concentration in offspring female rats

**Figure 3:**
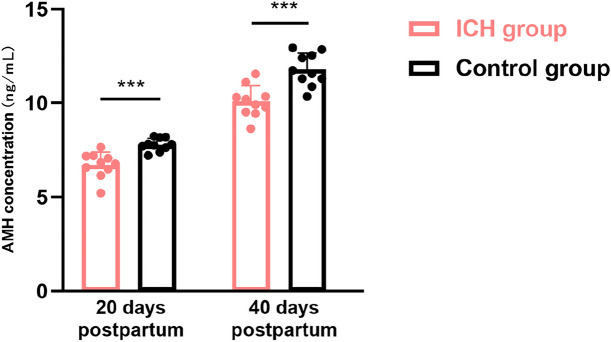
Comparison of Anti-Müllerian Hormone (AMH) levels in female rats in the offspring of ICH and control groups (ICH group n = 10, control group n = 10). Data are presented as mean and standard deviation. *p* values were calculated using unpaired t test. ***p < 0.001. ICH: intrauterine chronic hypoxia.

Owing to the role of AMH as an indirect marker of the ovarian reserve, this study evaluated serum AMH levels in two groups of animals. Offspring female rats on postnatal day 5 were not detected owing to difficulties in blood sampling. AMH concentrations on postnatal days 20 and 40 in the ICH group (6.72 ± 0.68 ng/mL; 10.09 ± 0.84 ng/mL) were significantly lower than those in offspring female rats in the control group (7.79 ± 0.33 ng/mL; 11.79 ± 0.87 ng/mL) (t = 4.499 and t = 4.464, both *p* < 0.001) (Fig. 3).

## Discussion

Notably, ICH is one of the most commonly observed adverse environmental factors in pregnancy that can inhibit the invasion of trophoblast cells into the small uterine spiral arteries, leading to increased maternal blood pressure, placental insufficiency, reduced nutrient and oxygen delivery, and intrauterine growth restriction of the foetus^19^. Therefore, in this study, an ICH model was established from the 4th day of pregnancy until full term in female rats of the maternal generation. Blood gas analysis showed that the blood PaO_2_ and SaO_2_ of the rats in the ICH group decreased significantly (*p* < 0.05), suggesting hypoxaemia, and there was no significant difference in the blood PaCO_2_ and pH values (*p* > 0.05), indicating that the pregnant rats did not show obvious acidosis and carbon dioxide retention. Therefore, the model resulted in the pregnant rats being subjected to hypoxic stress without being affected by other factors, and this is suitable for the unifactorial study of hypoxia. Adverse outcomes, such as death or premature birth, were not observed in the pregnant rats, proving that the ICH model established using this method is safe and feasible. The results of this study further confirmed that ICH caused intrauterine growth restriction, low birth weight of the offspring, and the phenomenon of ‘catch-up growth’ after birth, and there was no significant difference in the BWs of the offspring between the two groups on postnatal day 20 (*p* > 0.05).

ICH may be programmed to control and induce adaptive changes in foetal tissues and organs to adapt to the hypoxic intrauterine survival environment, which persists for a long time. When the environment changes after birth, these adaptive changes may no longer be favourable to growth and development, inducing a series of associated diseases. The ovarian reserve of primordial follicular oocytes is formed during intrauterine development and represents the entire supply of oocytes required to sustain female fertility. Inadequate oxygen to the foetus results in intrauterine growth restriction and may affect gonadal development in the offspring, with potential implications for fertility.

We showed that the number of primordial follicles in the ovaries of female offspring in the control and ICH groups gradually decreased from postnatal day 5 to postnatal day 40, consistent with the expected age-related decrease in the number of primordial follicles from birth to puberty. The number of follicles at all levels and the total number of follicles in the ovaries of female rats in the ICH group were significantly reduced on postnatal days 5 and 20 compared with those in the control group (both *p* < 0.05). On postnatal day 40, the only differences observed between the two groups of animals were in the number of primordial follicles and the total number of follicles (*p* < 0.001 and *p* = 0.002, respectively); there were no differences in the numbers of follicles at other developmental stages (*p* all > 0.05). Because intrauterine growth restriction can affect the ovarian reserve function in the early developmental stages of offspring, the presence of compensatory mechanisms before puberty may allow offspring to retain their fertility.

We further found that the number of primordial follicles of female rats in the offspring of the ICH group was 27.1% lower than that of the offspring of the control group on postnatal day 5, 40.0% lower on postnatal day 20, and 41.6% lower on postnatal day 40 (all *p* < 0.05). By contrast, there was a trend towards lower AMH concentrations in female offspring in the ICH group on postnatal days 20 and 40 than those in the control group, and the differences were statistically significant, suggesting that exposure to adverse factors in early life), e.g. intrauterine hypoxia, has a profound effect on the ovarian reserve, an effect that may become more pronounced as the offspring grow older. The low ovarian reserve identified in this study may be attributable to any or all of the following factors: decreased production of pre-granulosa cells (GCs) or oocytes during intrauterine ovarian development, increased apoptosis during oogenesis, or decreased follicle formation after the establishment of the primordial follicular reserve after birth. By contrast, there was no difference in the number of primary, secondary, or antral follicles between the two groups 40 d after birth, which may be attributed to a decrease in granulosa cell apoptosis. Alternatively, this phenomenon may be attributed to the increase in primordial follicle activation, where primordial follicles continued to move from the quiescent pool into the growth pool to support the increase in the number of developing follicles in the ICH group. Thus, only a decrease in the number of primordial follicles was observed, with no difference in the number of follicles in the remaining levels, which is consistent with the observed decrease in ovarian reserve of primordial follicles.

By using the digital gene expression profiling technique, this study revealed that three genes (MKi-67, Pcna, and Tk-1) involved in cell proliferation were increased in the ovaries of hypoxic zygote females on postnatal day 5, which may be associated with an increase in primordial follicle activation in support of growing follicles and sinusoidal follicle number growth in the ICH group. By contrast, Top2 expression was downregulated on postnatal days 20 and 40. Tk-1 expression was also downregulated on postnatal day 40, which may reflect a low primordial follicle activation rate on postnatal day 40. A possible reason for this result is that the ICH group had reached a certain number of growing follicles and antral follicles at a later stage, as in the control group; thus, proliferation was reduced. The same significance may be attributed to the modification of genes belonging to the IGF system; the decrease in IGF-1R expression and the parallel increase in IGFBP-3 expression in the ICH group on day 40 predicted a decrease in the activity of IGF-1, one of the most important growth factors in the ovary, suggesting a possible role for the insulin/IGF pathway; however, comparing postnatal days 5, 20, and 40 is not sufficient to support the insulin/IGF-1 signalling pathway. The IGF family comprises three classes of protein polypeptides: insulin (INS), IGF-1, and IGF-2. IGF-1 is one of the most important growth factors in the ovaries. It plays an important role in prenatal and postnatal growth and development of daughter rat and specifically binds to IGF-1R through autocrine, paracrine, and endocrine forms, exhibiting its effects of promoting cell proliferation, differentiation, and inhibiting apoptosis. IGF-1R expression was downregulated on postnatal day 40, indicating that the ovaries of female rats in the ICH group stopped proliferating and differentiating.

Usselman et al.^20^ showed that low oxygen concentrations can lead to ovarian microvascular dysfunction and the inhibition of the vasodilatory response, leading to ovarian insufficiency. Therefore, three genes related to ovarian angiogenesis were dysregulated in hypoxic offspring rats. The expression of TGF-β2 was upregulated on day 20 in offspring rats of the ICH group, and we hypothesised that this factor was also involved in apoptosis control during postnatal life. Wood and Sills found that the ovarian follicular fluid presented with hypoxia saturation might amplify various local cell death pathways and promote apoptosis and necrosis^21^. Consistently, the expression of the gene for casp-3 was upregulated in female rats in the ICH group compared with that in the control group on postnatal day 5. By contrast, the expression of casp-9 was significantly downregulated, and apoptosis was reduced in the offspring of the ICH group on day 40, consistent with the observation in this study that there was no difference in the number of primary, secondary, or antral follicles between the two groups on postnatal day 40.

This study had some limitations. First, the onset and progression of puberty and late adulthood and the distribution of sex steroid hormones were not assessed in the experimental animals. Second, although the findings suggest that intrauterine hypoxia affects the fertility of female offspring, robust fertility tests were not performed, and the fertility of rats with intrauterine growth-restricted offspring has not been assessed by verifying mating outcomes. Thus, further breeding studies involving multiple mating are required to definitively determine whether the reduced ovarian reserve reported in this study translates to a shortened fertile lifespan. Therefore, whether the normal number of growing follicles at puberty in hypoxic foetal offspring reflects the full reproductive potential and, most importantly, whether it is maintained throughout the reproductive life span remain unclear. Third, the results of this study must be interpreted with caution because of the limited amount of tissue available to validate gene expression data at the protein level. Genes involved in cellular processes are affected at all ages, which may imply long-term genetic alterations. However, further analyses are necessary to elucidate the effects of foetal intrauterine hypoxia on ovarian function and fertility in late adulthood.

In conclusion, the present study demonstrated that ICH-induced intrauterine growth restriction affects the number of follicles in female offspring, specifically targeting primordial follicles, suggesting an effect of intrauterine hypoxia on the ovarian primordial follicular pool. Exposure to chronic hypoxia during foetal development leads to an increase in primordial follicle activation to support the growth of follicle numbers in the hypoxic group, with continuous depletion of the primordial follicle pool, a decrease in ovarian reserve, and folliculogenesis continuing until the primordial follicle reserve is exhausted and menopause occurs. The genes involved in cellular processes were affected at all ages, indicating long-term genetic alterations. Differences in the expression profiles of miRNAs in the ovaries of female offspring in the ICH group compared with the control group suggest that miRNAs with significant differences in expression in the ovarian tissues of female offspring from the two groups may be involved in the pathogenesis of DOR. ICH affects ovarian function and fertility, and a long-term longitudinal study is still necessary to investigate whether the entire reproductive lifespan is affected.

## Methods

### Establishment of an animal model of ICH

Twenty-four Sprague–Dawley (SD) 8- to 9-week-old male and female rats, each weighing 160–200 g, with no health concerns or history of mating, were purchased from Shanghai Slaughter Laboratory Animals Limited Liability Company (Licence No.: SCXK (Shanghai) 2017-0005, Certificate of Conformity No.:2007001105427). The animal study protocol was approved by the Ethics Committee of the Second Affiliated Hospital of Fujian Medical University (2020-395). All methods were conducted in accordance with the relevant guidelines and regulations, and we followed the recommendations outlined in the ARRIVE guidelines for conducting research on animals The Laboratory Animal Center of Quanzhou University of Higher Medical Sciences in Fujian Province provided feeding and observation sites (Fujian Provincial Department of Science and Technology, Licence No.: SYXK (Min) 2016-0001, Laboratory Animal Management Committee of Quanzhou University of Higher Medical Sciences, Certificate of Conformity: No. 2012001). The rats that were adapted to feeding for 1 week, with no adverse reactions, and with normal diets, drinking water, and activities (20 females and males each), were included in the experiment. The male-to-female ratio was 1:1, and the rats were placed in the same cage at approximately 5:00. The rats were mated in the same cage at approximately 17:00. Rats with vaginal plugs detected on the white paper of the substrate early in the morning of the following day were considered pregnant and comprised the experimental group; this day was called gestation day 1. Pregnant rats were randomly divided into the control group (n = 8) and the chronic intrauterine hypoxia (ICH) group (n = 12).

The ICH group modelled intrauterine hypoxia as described by Williams et al.^22^. They were placed into a low-pressure hypoxia chamber on the 4th day of gestation. In the chamber, the air was continuously mixed by a small fan; the oxygen concentration in the chamber was continuously monitored by an oxygen concentration monitor, which adjusted the flow rate of oxygen and nitrogen in real time such that the oxygen concentration in the chamber was controlled to be 10% ± 0.1%; the carbon dioxide and water vapour in the chamber were absorbed by calcium chloride and sodium lime, respectively; and the carbon dioxide concentration in the chamber was always less than 3%. Hypoxia was applied twice daily, in the morning and afternoon, for 4 h each time until the 21st day of gestation. In this study, this hypoxic pregnancy model did not reduce food intake in pregnant rats^23^. Each rat in the control group was housed in an equal-volume chamber with normal oxygen levels during the same period and not treated with hypoxia. During gestation, both groups were fed a normal diet, separated into cages for labour, and delivered naturally.

On the first day after  4 h of hypoxia, four pregnant rats from each of the ICH and control groups were randomly selected for arterial blood gas analysis to confirm the success of the model.

### Grouping of experimental animals

Pregnant rats delivered spontaneously between the 19th and 23rd day of gestation. Sex was determined according to the distance between the genitals and the anus, and the female offspring were retained for further rearing according to our experimental needs; the weights of the newborn pups were recorded once per week. Newborn pups were allocated to females for feeding, with a maximum of six pups per litter. Those under the intrauterine growth retardation model, whose birth body weight ([BW]) was more than two standard deviations below the mean BW of the control litter, were included in the ICH group. Thirty female rats from each of the ICH and control groups were divided into six subgroups (n = 10) by using a random number table. The ICH groups were ICH-A, postnatal day 5 (neonates, end of follicular pooling); ICH-B, postnatal day 20 (juveniles, emergence of the first large sinusoidal follicle and medullary follicular atresia); and ICH-C, postnatal day 40 (puberty, first ovulation)^24^. The control groups were Control-A, 5th day after birth; Control-B, 20th day after birth; and Control-C, 40th day after birth. On the 21st day after birth, both groups were separated from their mothers and fed standard diets in litters of 3–5 rats.

### Histologic analysis and follicle count

At postpartum days 5, 20, and 40, offspring rats from the ICH group and the control group (n = 10 each) were euthanized by cervical dislocation. The ovaries obtained from the surgery were fixed, dehydrated, and embedded in paraffin. They were then cut into consecutive sections with a thickness of 5μm using a microtome without gaps. These sections were mounted on microscope slides and left overnight at a temperature of 37 °C. The ovaries were then removed from the cervical spine, and the rats were euthanized by cervical spinal dislocation.

**Figure 4:**
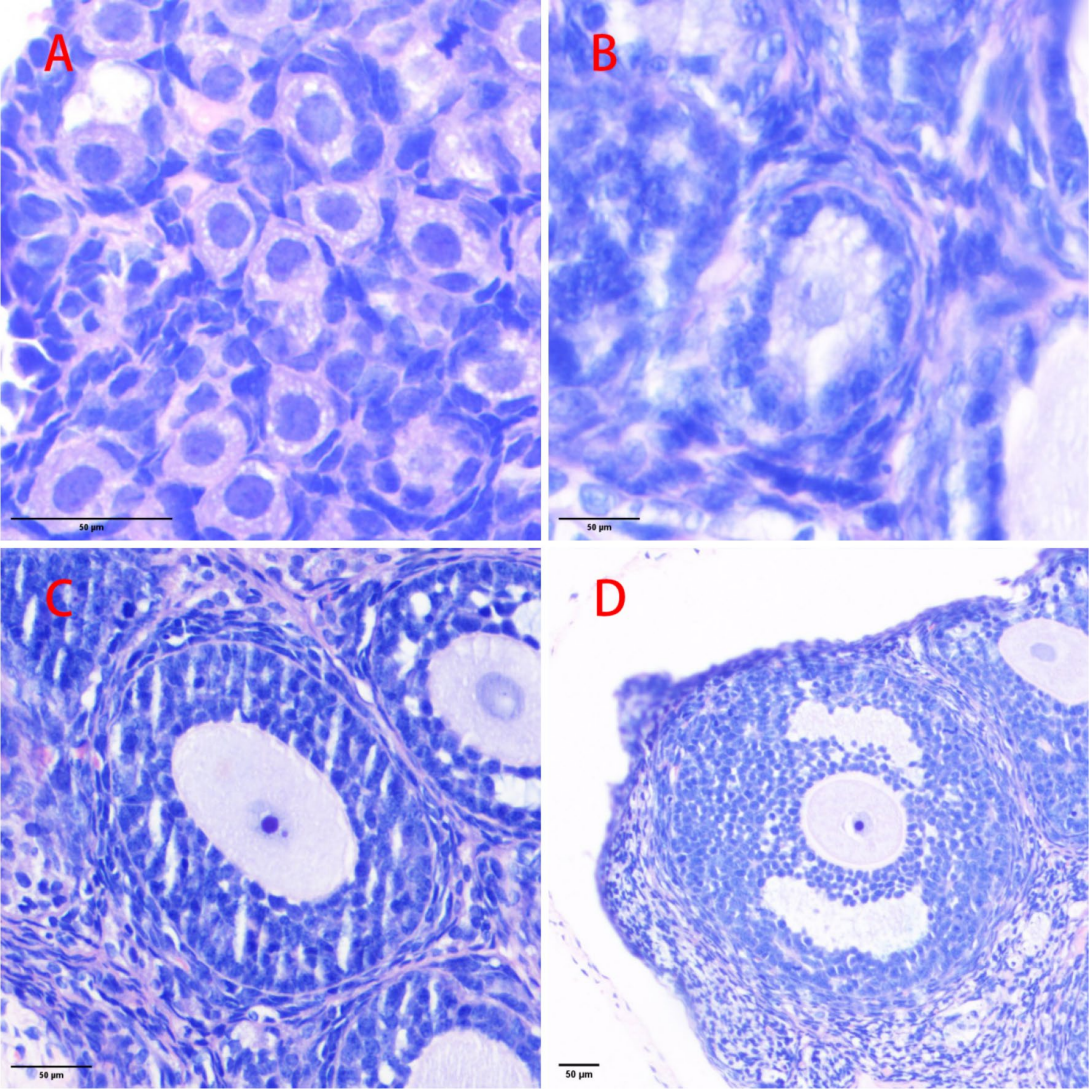
Morphological classification of ovarian follicles in offspring female rats. (**A**) Primary follicle (HE, ×400) with a flattened layer of pre-granulosa cells (GCs); (**B**) Primary follicle (HE, ×200) with two or more cuboidal GCs and at most one complete layer of cuboidal GCs; (**C**) Secondary follicle (HE, ×200) with at least two layers of GCs but no sinusoidal lumen; (**D**) Sinus follicle (HE, ×100) with visible follicular lumen^28,29^.

The ovarian sections were stained with haematoxylin and eosin. Three sections from each ovary were selected for follicle counting at three points (25%, 50%, and 75%) of the ovary^25^, and the total number of follicles was estimated. To avoid double counting the follicles, we counted only follicles with visible oocyte nuclei^26^ (Fig. 4). Sections were numbered differently and read in a double-blind manner by two physicians with comparable experience who independently evaluated the same section and compared the counts. The results showed an interobserver agreement of >90%.

Estimation of ovarian volume: the ovary approximates an elongated ellipsoid^27^; thus, the ovarian volume (mm^3^) was estimated using the ellipsoidal formula 4/3πab^2^, where ‘a’ is the length of the entire ovary by multiplying the total number of sections (n) by 0.005 (representing the thickness of each section), and πb^2^ is the area in the middle of the ovary (Am). Thus, ovarian volume (v) was 4/3 × Am × n × 0.005.

### RNA isolation and cDNA synthesis

Total RNA was extracted from the ovaries of 10 rats in the ICH group and 10 rats in the control group at postpartum days 5, 20, and 40. RNA was extracted using a single-beam UV/Vis spectrophotometer. For generating cDNA, 1 μg of RNA was reverse-transcribed using a thermal cycler using a random hexamer in a total reaction volume of 20 μl. Finally, cDNA was synthesised using a cDNA synthesis kit.

### Digital gene expression profiling technology

Digital gene expression profiling technology was used to compare and analyze the gene expression in the ICH group and the Control group animals (n = 10 each) at postpartum days 5, 20, and 40. Gene expression is presented as relative expression (calculated using the fold-change method [2-ddCT]). TaqMan Low Density Array (TLDA) analysis was performed using the TaqMan Gene Expression Premix.

TLDA Cards (P/N 4342259, ABI, Hilden, Germany) were used for comparative gene expression analysis of the ICH and control groups, according to the manufacturer's protocol. In brief, the TLDA Card analysed the expression of a set of 96 genes at a time based on TaqMan chemistry; the TLDA Card comes pre-loaded with 96 TaqMan gene expression assays, and six endogenous controls were specified for normalisation. Gene expression was normalised to the average of five of the six endogenous controls (actin β, β-2-microglobulin, connexin β1, eukaryotic translation elongation factor 11, and glyceraldehyde-3-phosphate dehydrogenase) for the same sample (dCt), selected based on their stability. Data from one randomly selected animal were used as calibrators (ddCT) and normalised to the data from all other animals. Finally, gene expression was expressed as relative expression (calculated using the fold change [2-ddCT] method). When TLDA assays were performed, TaqMan Gene Expression Premix (P/N 4369510, Applied Biosystems, MA, USA) was used.

### Measurement of serum Anti-Müllerian Hormone concentration

Anti-Müllerian Hormone (AMH) serum levels in the ICH and control groups (n = 10 each) were measured on days 5, 20, and 40 after the birth of the offspring by using the Gen II ELISA kit (reference A79765) and a calibrator kit (Beckman Coulter) according to the manufacturer's instructions. The lower detectable limit was 0.08 ng/ml, and the intra- and inter-batch coefficients of variation, that is, the low and high levels of the standard curves) were 4.3% and 9.8% and 1.4% and 4.3%, respectively.

### Statistical analysis

The data were analysed using the SPSS 26.0 statistical software package: the measurement information is expressed as mean ± standard deviation (x ± s). The difference between the two groups was compared using the independent samples t test, and the difference was considered statistically significant at *p* < 0.05.

## Conclusion

We showed that ICH decreased the ovarian reserve function of female rat offspring and programmatically regulated the differential expression of miRNAs in the ovaries of female offspring, which was related to the functions of cell proliferation, control, apoptosis, and vascularisation, which may be the mechanism for the development of ICH-induced decrease in ovarian reserve function.

## Data Availability

The datasets used and analysed during the current study available from the corresponding author (Guorong Lyu) on reasonable request.
